# A Computational Approach to Increasing the Antenna System’s Sensitivity in a Doppler Radar Designed to Detect Human Vital Signs in the UHF-SHF Frequency Ranges

**DOI:** 10.3390/s25103235

**Published:** 2025-05-21

**Authors:** David Vatamanu, Simona Miclaus

**Affiliations:** 1 Doctoral School of Electrical Engineering, Technical University of Cluj-Napoca, 400020 Cluj-Napoca, Romania; vatamanu.david@forter.ro; 2 Department of Communication, IT and Cyber Defense, “Nicolae Balcescu” Land Forces Academy, 550170 Sibiu, Romania

**Keywords:** human respiration simulation, Doppler bio-radar, microwaves, S21 phase change, multiple regression

## Abstract

In the context of Doppler radar, studies have examined the changes in the phase shift of the S_21_ transmission coefficient related to minute movements of the human chest as a response to breathing or heartbeat. Detecting human vital signs remains a challenge, especially when obstacles interfere with the attempt to detect the presence of life. The sensitivity of a measurement system’s perception of vital signs is highly dependent on the monitoring systems and antennas that are used. The current work proposes a computational approach that aims to extract an empirical law of the dependence of the phase shift of the transmission coefficient (S_21_) on the sensitivity at reception, based upon a set of four parameters. These variables are as follows: (a) the frequency of the continuous wave utilized; (b) the antenna type and its gain/directivity; (c) the electric field strength distribution on the chest surface (and its average value); and (d) the type of material (dielectric properties) impacted by the incident wave. The investigated frequency range is (1–20) GHz, while the simulations are generated using a doublet of dipole or gain-convenient identical Yagi antennas. The chest surface is represented by a planar rectangle that moves along a path of only 3 mm, with a step of 0.3 mm, mimicking respiration movement. The antenna–target system is modeled in the computational space in each new situation considered. The statistics illustrate the multiple regression function, empirically extracted. This enables the subsequent building of a continuous-wave bio-radar Doppler system with controlled and improved sensitivity.

## 1. Introduction

The non-contact monitoring of human vital signs, specifically respiration and heart rate, presents significant advantages in healthcare, rescuing, and sensing applications, enabling continuous and unobtrusive monitoring. Doppler radar technology, operating in various frequency bands, offers a promising approach for this purpose because it is capable of accurately detecting minute movements associated with these vital functions [1,2]. To enhance the detection sensitivity, numerous studies have explored different antenna designs that utilize diverse configurations, such as flexible antennas [2,3], horn antennas [4], patch antenna arrays [5], microstrip antennas [6,7], leaky-wave antennas [8], and frequency selective surface antennas [9]. These designs exhibit varying performance characteristics in terms of their gain, directivity, bandwidth, and fabrication complexity [10,11]. The choice of operating frequency also profoundly affects the degree of sensitivity; higher frequencies potentially offer an improved resolution but can suffer from increased attenuation and susceptibility to environmental factors [1,8,12]. Furthermore, the interaction between the electromagnetic waves and the target (the human chest) is influenced by factors such as the tissues’ dielectric properties and the antenna–target distance [13].

The performance of a Doppler radar system for the detection of vital signs is intrinsically linked to the antenna characteristics and signal processing techniques. While higher frequencies like W-band (75–110) GHz offer high-resolution measurements [1], their practical implementation faces challenges related to the signal-to-noise ratio (SNR) and environmental sensitivity [13]. Optimizing the antenna design to maximize the signal strength and minimize the noise interference is paramount [13]. Efficient signal processing algorithms are needed to isolate subtle Doppler shifts caused by respiration and heartbeat from background noise and artifacts, with the choice of technique being highly dependent on the system design, operating frequency, and environmental conditions [14]. The selection of the operating frequency is critical, influencing both the system’s sensitivity and its applicability. Higher frequencies provide improved spatial resolution but may suffer from increased attenuation [1,8,15], while lower frequencies, such as 2.45 GHz [12], offer a balance between penetration and resolution. The feasibility of detection, even through physical obstacles, has been demonstrated [16], underscoring the importance of considering the specific application environment [10].

The following grouped sub-sections provide an overview of the current state of knowledge regarding the essential points to consider when designing a proper Doppler radar system with continuous wave (CW) that is designed to detect vital signs.

***A. Antenna Design and Performance for Vital Sign Detection:***

The development of accurate and reliable non-contact vital sign monitoring systems has driven extensive research into the optimization of antenna designs for Doppler radar applications. This overview describes a variety of antenna configurations, each with its own characteristics and trade-offs.

At lower frequencies, systems utilizing microstrip antennas have been explored for their cost-effectiveness and ease of integration [6,7]. For instance, Vatamanu et al. [7] compared microstrip patch and Sierpinski fractal antennas and found that fractal designs can offer improved bandwidth and multi-band capabilities, which are advantageous for vital sign detection. Other configurations, including patch antenna arrays and frequency selective surface antennas, have been studied for their high accuracy in remote vital sign monitoring [5,9].

Investigations into microstrip array modules [17] and leaky-wave antennas [8] at different frequencies (such as 24 GHz and 60 GHz) have revealed unique benefits and trade-offs, balancing performance and practicality. At medium to high frequencies, 24 GHz flexible antennas have shown promise for their conformability to the human body, which is essential for vital sign monitoring. Kathuria and Seet [2] demonstrated the effectiveness of a 24 GHz flexible antenna, emphasizing the benefit of flexibility. Their subsequent work [3] with a flexible Liquid Crystal Polymer (LCP) antenna array further improved the performance, especially concerning the radiation pattern effects.

In the W-band (around 94 GHz), antenna designs have advanced to include substrate integrated waveguide (SIW) slot antennas that provide an enhanced gain and bandwidth [18]. Researchers have also developed integrated tapered array antennas [19] within this band to improve the resolution and accuracy in radar-based monitoring. Furthermore, 60 GHz systems have been developed, including compact, integrated micro-radar systems-in-package for non-contact vital sign detection [15] as well as miniature circularly polarized continuous wave (CW) Doppler radar systems [20]. Additionally, a dual circularly polarized circular patch antenna has been utilized to create miniature Doppler radar systems [21].

Overall, these diverse antenna designs highlight the ongoing efforts to strike a balance among the performance, cost, and ease of integration. Gouveia et al. [10] underscored the importance of optimized antenna configurations, demonstrating a clear link between the antenna design and bio-radar system sensitivity.

Finally, comprehensive investigations into various antenna design techniques for vital sign monitoring [11] provide valuable insights into the broad spectrum of available approaches, emphasizing the continuous evolution in this research field.

***B. System Performance and Signal Processing:***

The performance of a Doppler radar system for vital sign detection depends heavily on both the antenna characteristics and the effectiveness of the signal processing techniques used to extract subtle movements such as respiration and heartbeat.

At the lower end of the frequency spectrum, systems have employed various design choices to improve the detection capabilities. El-Samad et al. [14] compared single and two-antenna vector network analyzer (VNA) setups for heartbeat rate extraction, demonstrating that a two-antenna system provides a better SNR and reduced sensitivity to environmental noise. This comparison underscores the importance of system design choices in optimizing performance. The selection of signal processing methods, including filtering, phase detection, and spectral analysis, plays a crucial role in enhancing the accuracy and reliability of vital sign measurements, often depending on the specific system design, operating frequency, and environmental conditions.

In studies examining higher frequencies, Kim and Jeong [1] demonstrated the potential of a W-band Doppler radar for non-contact respiration and heartbeat measurement, highlighting its capability for high-resolution detection at these frequencies. However, the practical implementation at such high frequencies presents challenges, such as a low SNR and sensitivity to environmental factors, which complicate the real-world applications.

The importance of careful antenna design in optimizing signal strength and reducing noise interference was further emphasized by Das et al. [13], who evaluated a non-contact vital signs sensor’s antenna performance. Their work advocates for a holistic approach, considering both antenna characteristics and signal processing techniques to enhance the system’s robustness.

Given the subtle nature of the signals involved, advanced signal processing algorithms are usually required to isolate the Doppler shifts caused by respiration and heartbeat from background noise, artifacts, and clutter. Various techniques have been developed for this purpose, including resolution enhancement methods for monitoring multiple subjects [22], bandwidth-limited FMCW radar processing [22], environmental noise filtering [23], and an energy aggregation analysis for multi-person localization and vital signs detection [24]. Other approaches involve combined CW and FMCW radar systems [25], the mitigation of proximity stationary clutter [23], and specialized signal processing algorithms for the accurate extraction of vital signs from mm-wave radar data [26].

This progression from lower to higher frequencies and more complex processing techniques highlights the evolving efforts to improve the sensitivity, accuracy, and environmental robustness of vital sign detection systems. Overall, this underscores the need for an integrated system in which antenna design and sophisticated signal processing work together harmoniously—particularly as operating frequencies increase—to achieve optimal performance in real-world scenarios.

***C. Frequency Considerations and Applications:***

The choice of operating frequency is a critical parameter in the design of Doppler radar systems for vital signs detection, as it significantly influences both sensitivity and practical application considerations.

At lower frequencies, such as UHF and SHF bands, the systems typically prioritize the penetration depth and portability. For example, low-frequency CW radar systems are recommended for their low power consumption and ease of portability [27]. Bo et al. [12] explored the use of 2.45 GHz, which offers a balance between penetration and resolution, making it suitable for applications requiring less sensitivity but broader coverage.

At higher frequencies, such as 24 GHz and 60 GHz, the systems demonstrate improved spatial resolution, allowing for the detection of smaller movements associated with vital signs [1,8,15]. These mm-wave FMCW radar systems have shown excellent performance in this regard, with [22,28] confirming their suitability for precise measurements. Additionally, ultra-wideband (UWB) radar sensors have been employed to effectively detect respiration and heart rate through obstacles [29].

Some studies, like that of El-Samad et al. [16], have demonstrated the feasibility of heartbeat detection even behind obstacles such as walls, using continuous wave (CW) Doppler radar at certain frequencies. This highlights the importance of considering the specific environment and the trade-offs between resolution and obstacle penetration: higher frequencies tend to experience greater attenuation but offer finer spatial details, while lower frequencies penetrate obstacles better but with a lower resolution.

In this context, the use of 2.45 GHz WiFi bands can be suitable for passive sensing applications, offering a compromise between penetration and resolution [30]. Conversely, environments requiring high resolution and precision benefit from higher frequencies, such as 24 GHz and 60 GHz FMCW radars, which have delivered outstanding results in various studies [22,28]. These frequencies are particularly effective in cases in which the detailed measurement of respiratory and cardiac movements is vital.

Evaluations by Gouveia et al. [10] further emphasize that selecting the optimal frequency band depends heavily on the particular application requirements and system design considerations. Therefore, determining the ideal operating frequency involves balancing factors such as the penetration depth, sensitivity, resolution, power consumption, and environmental conditions.

In the present study, the choice of the UHF-SHF frequency ranges is justified by the need for a sensitivity increase of a CW Doppler bio-radar, while bearing in mind the necessity of providing some penetration depth, since sensing the vital signs behind obstacles (like walls and doors) was also a later objective of our work.

Our analysis, performed through simulations using CST Studio Suite, focused on a system employing two identical antennas, which enabled a controlled investigation of the influence of antenna characteristics and other key parameters on the detection sensitivity. Specifically, we examined the impact of the operating frequency, the antenna gain and radiation pattern, the electric field intensity at the chest surface, the transmission distance, and the dielectric properties of the target material. The sensitivity was assessed by quantifying the phase shift pathway of the transmission coefficient (S21) while a simulated chest model underwent a 3 mm displacement in 10 steps, mimicking respiratory movement. Our central hypothesis was that the antenna gain and directivity would be the most significant parameters affecting the sensitivity of detecting respiratory and cardiac movements. By systematically varying these parameters, we aimed to establish design guidelines for maximizing the phase shift of the S21 parameter, thereby improving the sensitivity of vital signs detection and the resolution of the quantifications.

## 2. Materials and Methods

A rigorous numerical approach was used to investigate the influence of the antenna parameters on the sensitivity of a CW Doppler radar system for the non-invasive detection of subtle motions associated with human breathing. The study was performed entirely by a computational simulation using the Simulia CST Studio Suite [31]. The numerical analysis was focused on the evaluation of the S21 transmission coefficient’s phase shift variation in response to small changes in the position of a simulated target initially located at a precise distance from the antennas.

The simulated system configuration involved two identical antennas placed parallel to one another in a free-space environment. In front of the antennas, at a distance of 500 mm, a reflective planar target of the dimensions (100 mm × 100 mm) was placed. This target was initially modeled as a Perfect Electric Conductor (PEC) to reproduce the ideal reflectivity conditions of a highly conductive object. Later, the material of the target was varied. The reflective target was moved incrementally in a direction perpendicular to the plane of the antennas over a total distance of 3 mm in constant steps of 0.3 mm each, thus simulating the fine motion associated with human breathing. This configuration allows a precise analysis of the sensitivity of the system to very small variations in the position of the reflecting object.

**Figure 1:**
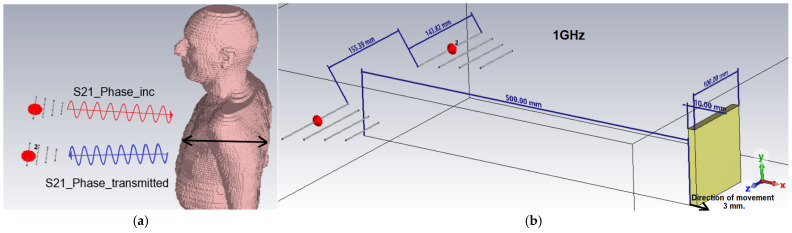
The simulation setup and basic principle used to evaluate the sensitivity of the CW Doppler radar system: (**a**) the principle of measuring the phase variation of the S21 parameter; (**b**) the geometry of the simulated Doppler radar system configuration in CST Studio Suite.

Figure 1a illustrates the fundamental principle of measuring the phase variation of the S21 parameter. It should be noted that the full human body shown in the figure was not used in the conducted simulations; its depiction provides a better understanding of the general concept of the measurement. The main idea is to measure how the electromagnetic wave transmitted from the first antenna travels through the space, interacts with the chest (due to breathing or heartbeat movements), and is received by the second antenna. The S21 parameter specifically refers to the transmission coefficient between the two antennas—that is, it tells us how much of the transmitted signal reaches the receiving antenna and what its phase shift is. The phase shift of S21 indicates how the distance or movement of the chest modulates the phase of the wave during transmission (not reflection). In contrast, reflection measurements (like those involving the S11 parameter) focus on signals that bounce back to the same antenna. Here, the interest is in the transmitted wave passing through the chest and being received at the other side, providing information about movement based on changes in the transmitted wave’s phase during this passage. Thus, this measurement is transmission-based, not reflection-based. Figure 1b details the exact geometrical configuration used in the CST Studio Suite simulation environment, including the precise location of the antennas with regard to the planar PEC target (yellow parallelepiped) as well as the exact dimensions and distances between the simulated system components. Thus, the 500 mm distance between the antennas and the target, the relative distances between the antennas, as well as the incremental direction of the target displacement (explicitly marked by the black arrow) are clearly indicated. This detailed representation supports the accuracy of the reproduction of the experimental conditions and validates the consistency of the parameters used in the numerical analysis.

Two distinct types of antennas were used in the numerical simulations performed: Yagi antennas and dipole antennas. These were designed and optimized for the operating frequencies of interest: 1 GHz, 5 GHz, 10 GHz, 15 GHz, and 20 GHz.

The simulated Yagi antennas initially consist of four elements and are characterized by a high gain and a high directivity, making them suitable for applications in which sensitivity to fine changes in the target position is essential. Subsequently, in order to perform a detailed comparative analysis and to evaluate the impact of the gain increase on the radar system performance, five-element Yagi antennas were additionally designed for the same operating frequencies. This second group of antennas with one additional element provides a higher gain, thus allowing an evaluation of the direct influence of an increased gain on the sensitivity and detection performance. The dipole antennas, having an omnidirectional radiation pattern and a lower gain, were used as reference antennas to evaluate the impact of directivity on the performance of the CW Doppler radar system.

The specific characteristics of the simulated antennas are as follows:

Yagi antennas (4 elements): The maximum gain varies between 3.24 and 4.85 dBi, with the highest value at 1 GHz (4.85 dBi) and the lowest value at 10 GHz (3.24 dBi). The efficiency of these antennas is particularly high, ranging between 93.28% and 100% in the frequency range analyzed.

Yagi antennas (5 elements): The maximum gain varies between 4.27 and 6.66 dBi, with the highest value at 1 GHz (6.66 dBi) and the lowest value at 5 GHz (4.27 dBi). They have been designed to achieve a higher gain than the original 4-element antennas, allowing the direct comparison of results for the same frequency and antenna type.

Dipole antennas: The gain is significantly lower than that of the Yagi antennas, ranging from 1.08 to 2.11 dBi. The overall efficiency of the dipole antennas is also high, ranging between 94.95% and 100%.

**Figure 2:**
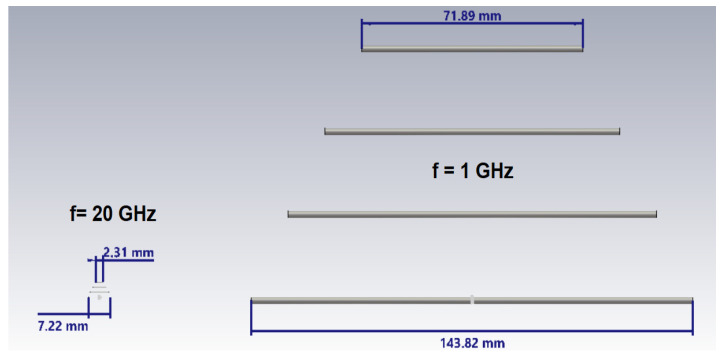
A dimensional sizing comparison between the Yagi antennas designed for 1 GHz and 20 GHz (grey colour: antenna elements; blue color: dimensional signs).

Figure 2 shows two Yagi antennas designed for the frequencies of 1 GHz and 20 GHz. The significant difference in their sizes clearly emphasizes the inverted relationship between the physical size of the antenna and its operating frequency, with the antenna designed for the low frequency (1 GHz) being considerably larger than the antenna designed for the high frequency (20 GHz).

Table 1 emphasizes the computational situations, underlining that all of the calculations were made in far-field conditions. At all five frequencies, the maximum dimensions of the two antennas—aperture *D* (*D*max_Dipole and *D*max_Yagi)—are indicated, together with the calculations of the starting limit of the far-field distance, *L*, (*L*-Far field Dipole and *L*_far field Yagi), given by the following relation: $L=2\times D^{2}/\lambda$, where λ is the wavelength.

**Figure 3:**
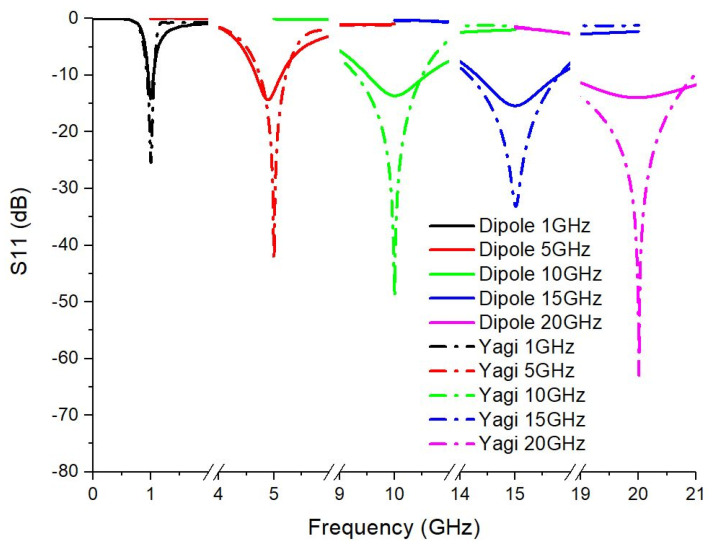
S11 reflection coefficients of simulated dipole and Yagi antennas at frequencies of interest.

To evaluate the impedance matching of each antenna at the frequencies used, the magnitude of the reflection coefficient S11 was analyzed. Figure 3 shows the overlapped curves of S11 for the dipole antenna and the Yagi antenna (with 4 elements).

For the dipole antennas, it is observed that the minimums of the S11 coefficient are well defined at each of the analyzed frequencies, indicating a good match of the antenna at these frequencies. For the Yagi antennas, the minimums of the S11 coefficient are more pronounced, suggesting a better matching at the selected frequencies, which may indicate a higher transmission efficiency. The S11 levels for the Yagi antennas are generally lower than for dipole antennas, which means that reflection losses are lower and, consequently, more energy is efficiently radiated.

**Figure 4:**
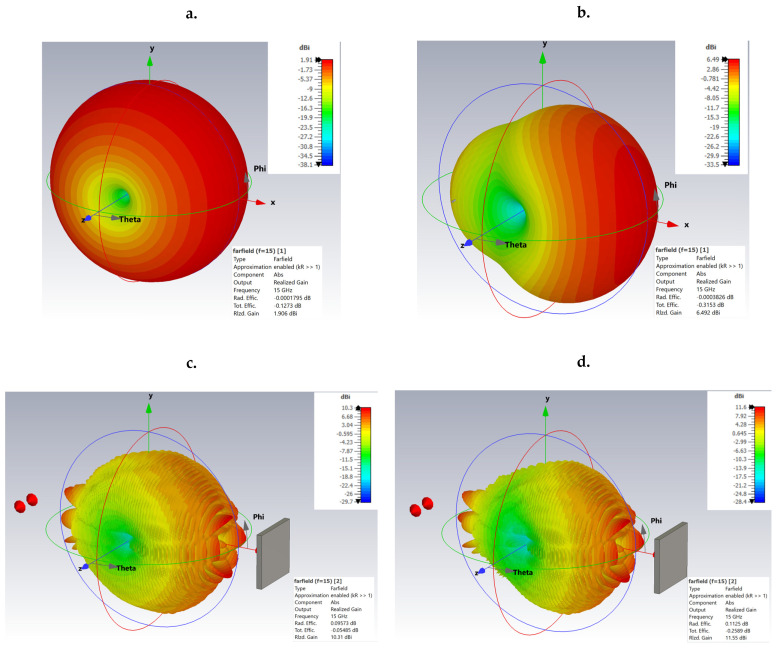
Three-dimensional radiation patterns of the simulated antennas: (**a**) 15 GHz dipole antenna; (**b**) 15 GHz Yagi antenna in free space; (**c**) 15 GHz Yagi antenna in the presence of a reflecting object; (**d**) 15 GHz Yagi antenna with an increased gain in the presence of a reflecting object.

Figure 4 shows the three-dimensional radiation patterns for four relevant situations: (a) 15 GHz dipole antenna—highlighting the omnidirectional distribution and low gain; (b) 15 GHz Yagi antenna—illustrating the high directivity; (c) 15 GHz Yagi antenna in the presence of the target—showing its effects on the radiation pattern due to reflection; and (d) 15 GHz Yagi antenna with a higher gain and in the presence of the same reflecting target—demonstrating the increased interaction and additional influences on the radiation due to the increased antenna gain.

**Figure 5:**
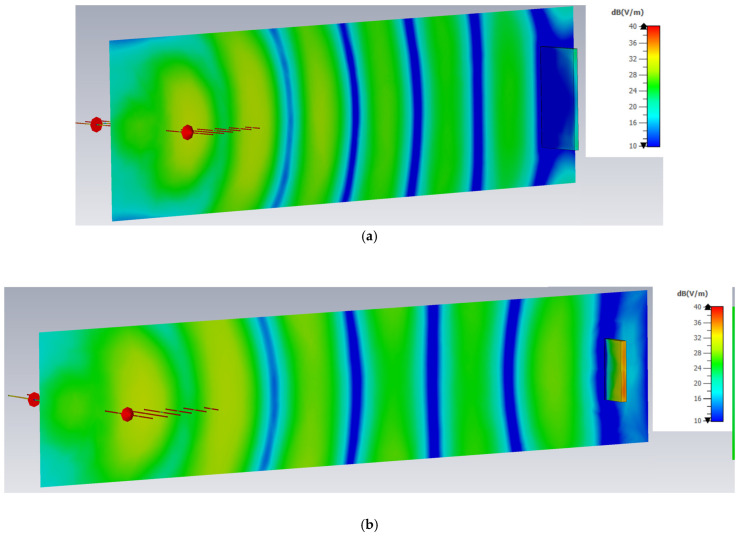
Planar wave-front impinging PEC target structure at lowest used frequency of 1 GHz: (**a**) distance antenna–target = 500 mm; (**b**) distance antenna–target = 1000 mm.

Regarding the use of computational situations in far-field conditions only, Figure 5 demonstrates the distribution of the field along the propagation path at the lowest frequency of 1 GHz in two cases: (a) when the distance between the antenna and the PEC target is 500 mm (as in all of the considered scenarios) and (b) when it is doubled to 1000 mm, for observing the difference in the planar wave-front image. In both cases, the target is present in front of the antenna system.

These results, therefore, provide insights into the selection and use of antennas in CW Doppler radar systems, highlighting how antenna parameters (gain, directivity, and efficiency) significantly influence the detection performance and detection sensitivity.

## 3. Phase Shift Variation Dependencies and Their Effect on the Detection’s Sensitivity

In this section, we present the results of the analysis of the following:

✓ The dependencies of the radar signal phase shift on the moving distance of the reflecting target (considered as PEC) for various frequencies for omnidirectional and directive antennas;
✓ The influence of the electrical parameters of the material target (metal and human tissues) upon the S21 phase shift variation;
✓ The cumulative effects of frequency, antenna gain, and incident electric field strength on the target’s surface upon phase shift changes by applying a multiple linear regression analysis.

The analysis refers to the simulations performed using the dipole and Yagi antennas for five different frequencies: 1 GHz, 5 GHz, 10 GHz, 15 GHz, and 20 GHz.

### 3.1. Phase Shift Effect of Frequency and Antenna Gain on a PEC Target

**Figure 6:**
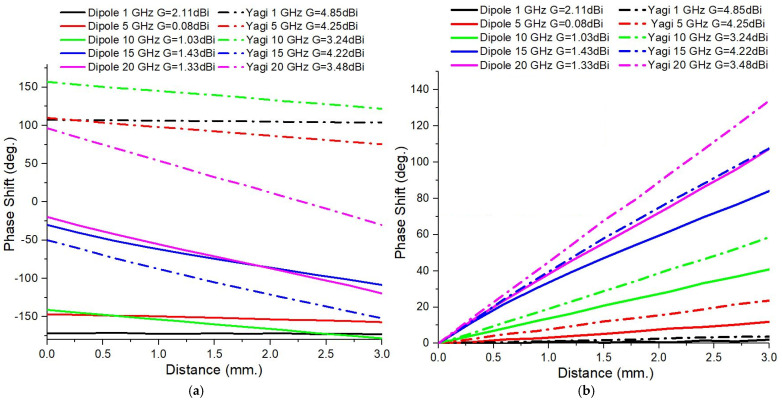
The S21 phase shift range corresponding to the PEC target displacement of 3 mm for the dipole and Yagi antennas, comparatively: (**a**) the raw results obtained from the simulations; (**b**) the corrected results obtained after extracting the phase shift change due to the different positioning.

Figure 6a compares the dependence upon the distance between the target and the antenna of the S21 phase shift for the two types of antennas. These cases correspond to the situation in which the Doppler target is made of a PEC. The S21 phase shifts during the target movement highly depend on the frequency, with Yagi antennas exhibiting more stable behavior at low frequencies and dipoles exhibiting more pronounced variations at higher frequencies.

In order to eliminate the phase shifts generated by the change of the position of the moving target, a phase correction was performed. Before normalizing the phase variations of the parameter S21, it was necessary to perform this correction. Practically, this refers to taking into account the difference in the geometrical path traveled by the electromagnetic waves between the transmitting and receiving antennas, which depends on the position of the PEC target position. This path difference contributes to the total phase variation of the received signal and must be eliminated from the calculations in order to compare the influence of the antenna gain and frequency on the phase change.

The general relationship between the geometrical path difference, Δ*L*, and the input phase shift, Δ*ϕ*, is given by the fundamental formula of plane wave propagation [32]:

(1) Δϕ=k⋅ ΔL

This formula reveals that an increase in the propagation distance causes an additional phase shift, Δ*ϕ*, proportional to the wavenumber, *k*. The new, corrected results are shown in Figure 6b. A linear increase in the S21 phase shift is observed as the PEC target moves. In all cases, the S21 phase shift is larger for the directive antenna, and increasing the frequency results in an enlargement of the differences in the S21 phase shift between the Yagi and dipole antennas. The S21 phase shift analysis revealed that the antenna type, operating frequency, gain, and directivity directly influence this parameter and, hence, the detection sensitivity. The wider phase variations also allow the movement to be detected from greater distances, making them suitable for applications such as breath monitoring and fine motion detection by electromagnetic analysis.

**Figure 7:**
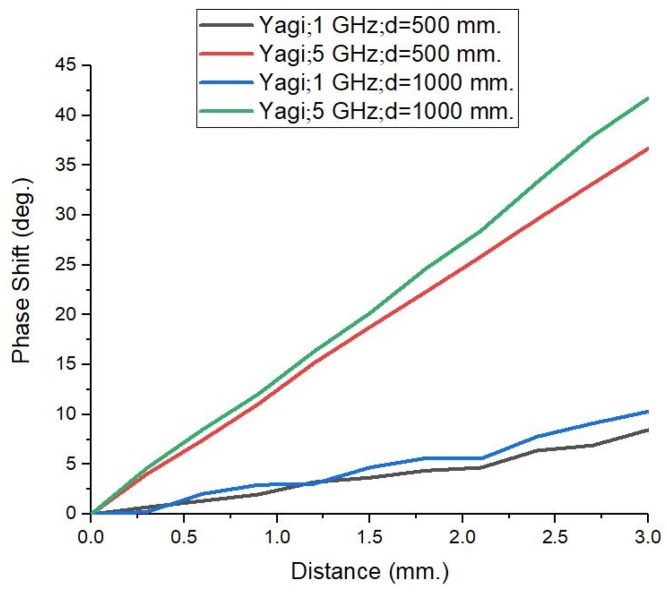
Proving the planar wave-front characteristics maintenance by showing the impact of doubling the distance between the antenna and the target, d, on the S21 phase shift change, while the solid angle of incidence was maintained (the target surface was made 4 times larger, while the distance was 2 times larger).

To assess the planar wave-front condition effect on the observed phase shifts, in Figure 7 we represent, comparatively, the effect of doubling the distance between the PEC target and antenna system from 500 mm to 1000 mm at the two lowest frequencies of 1 GHz and 5 GHz. In parallel, the target’s dimensions were enlarged to 400 × 400 mm^2^ in order to maintain the solid angle of incidence in both cases. Minute changes are observed, which fall within the expected standard deviation of the data.

### 3.2. Phase Shift Effect of Changing the Target’s Material Type

Another factor examined for its influence on the phase shift variations was the material from which the Doppler target is made. To investigate this aspect, the target’s material, located 500 mm in front of the antenna system, was changed from the PEC to the following:

(a) Copper;
(b) The following human tissues: muscle, skin, and fat.

This change of the dielectric parameters of the target allows the sensing of the impact of finite conductivity on wave propagation and reflection phenomena to be evaluated. The dielectric parameters of the human tissues at each frequency were extracted from [33].

**Figure 8:**
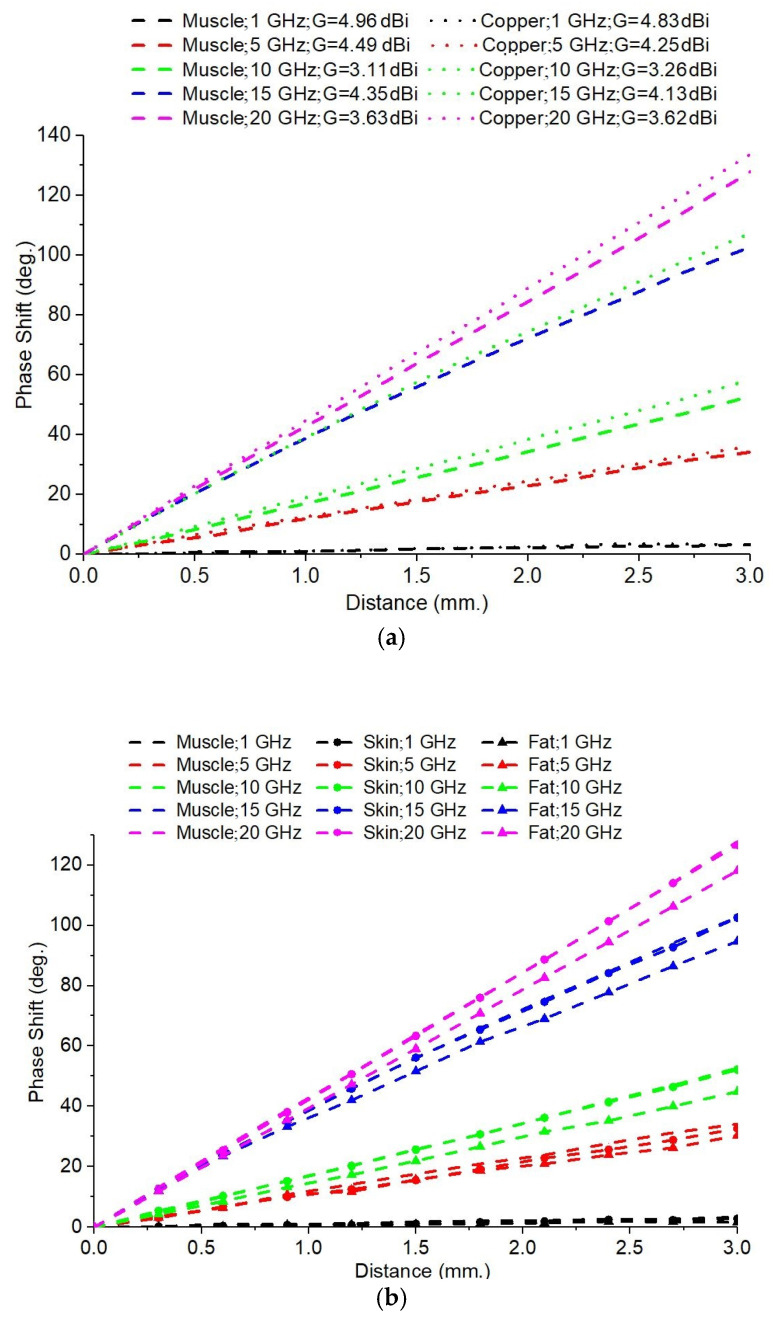
The influence of the target material on the S21 phase shift variation while the target moves: (**a**) comparison between muscle tissue and copper; (**b**) comparison between muscle, skin, and fat tissue.

Figure 8a shows the variation of the S21 phase with the displacement of the target for the two types of materials when using the 5-element Yagi antenna. The S21 phase shift was generally lower for muscle tissue compared to the copper target. This can be explained by the fact that muscle tissue has high losses and significantly lower conductivity than copper, leading to weaker reflection. For almost all of the frequencies, the slopes of the curves associated with muscle tissue are lower than those corresponding to copper. This suggests that lossy dielectric materials, such as muscle tissue, reduce the phase shift amplitude and delay the phase of the transmitted signal. For frequencies of 1 GHz, 10 GHz, 15 GHz, and 20 GHz, the differences between muscle tissue and copper are more pronounced at higher antenna gains, suggesting a stronger interaction between the radiated field and the reflecting material for more directive antennas.

In the case of the 4-element Yagi antennas, we can see that the phase shifts introduced by both copper and muscle tissue are relatively small. It is proven that, for a lower antenna gain, the impact of changing the reflective material is very small. At 1 GHz and 5 GHz, the differences between the curves associated with copper and muscle tissue are almost zero (probably in the computational error range). This indicates that, at lower frequencies, the losses in muscle tissue are less significant, and reflectance is still sufficiently effective. At 10 GHz, 15 GHz, and 20 GHz, a bit higher phase shift difference between the two materials is noted, suggesting that at higher frequencies, the dielectric effects become observable, and the wave experiences more attenuation in the presence of muscle tissue. The higher-gain 5-element Yagi antennas produce more pronounced phase shifts, if we compare the copper target with the muscle target, than the 4-element Yagi antenna. This emphasizes that the higher antennas’ gains are more sensitive to changes in the target’s material for the same frequency of the radar signal.

The simulations were similarly performed for the skin and fat tissues under the same conditions as in the previous case, and these yielded the results presented in Figure 8b, which correspond to the 5-element Yagi antenna. Again, we observe the small impact of a change in the tissue’s dielectric properties upon the S21 phase shift variation while the target is moving.

For both antenna designs (4-element and 5-element), the muscle tissue showed the largest phase shift at all frequencies analyzed, indicating that the muscle has the strongest influence on the signal propagation, most likely due to its higher conductivity, which results in a more pronounced reflection. The skin showed an intermediate effect; the values were closer to those of the muscle tissue than to those of the fat tissue. The fat tissue produced the smallest phase shift along the target movement, and its curves were almost zero-sloped at low frequencies. This suggests that the electromagnetic signal is less affected by the fat tissue, which can be explained by its lower permittivity and low electrical conductivity. At low frequencies (1 GHz, 5 GHz), the effect of the fat tissue on the phase shift variation was minimal, but it began to be visible at 10 GHz and above. It was also noted that the phase shifts increased significantly at higher frequencies (e.g., 15 GHz, 20 GHz).

The 5-element Yagi antenna (Figure 8b) provides a better separation of the results between the three tissue types. The higher gain results in more distinct differences between the three tissue types, making this setup more suitable for applications that require accurate phase shift tracking. Smaller differences between the tissues were observed in the 4-element Yagi antenna, which may indicate a lower sensitivity of this antenna to small phase shifts. The use of a higher gain antenna and a higher frequency (above 10 GHz) provided the best sensitivity, corresponding to differences between the tissue types during the respiration monitoring.

### 3.3. Phase Shift Cumulative Effects of Frequency, Antenna Gain, and Incident Electric Field Strength on the Target’s Surface: A Multiple Linear Regression Analysis

The impact of the frequency, antenna gain, and average electric field strength (E) on the phase shift and its variation with the planar target’s displacement was investigated. The modeling of these relationships was accomplished by linear regression.

The analysis was performed using three types of antennas: (a) a dipole antenna; (b) a Yagi antenna; and (c) a high gain Yagi antenna. Each of the antennas was placed, one by one, in front of the following materials of which the moving target was made: muscle, skin, fat, copper, and PEC. The calculations were performed at five distinct frequencies (1 GHz, 5 GHz, 10 GHz, 15 GHz, and 20 GHz).

The collected data included the calculation of the average phase shift per sweep, the maximum antenna gain, and the average electric field strength on the target’s surface.

**Figure 9:**
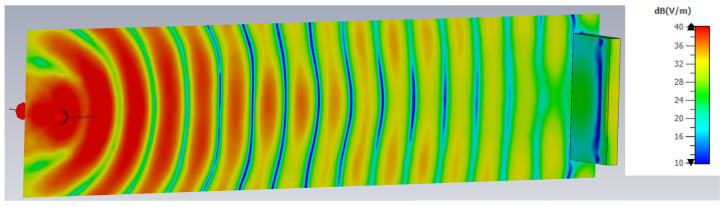
An example of the spatial distribution of the E-field strength generated by the antenna in the direction of the planar target (skin tissue) and on its surface at 5 GHz.

Figure 9 illustrates a representative example of the E-field strength distribution in air just tangent to the surface of the planar target, generated by the 5 GHz Yagi antenna on the skin tissue, showing the propagation of the electromagnetic wave and its interaction with the planar target positioned at a fixed distance from the antenna. This graphical representation was used to compute the average E-field strength on the target’s surface, and it was essential in performing the multiple linear regression analysis.

The phases of the analysis were as follows:

✓ Segmentation of the dataset into 11 subsets, each corresponding to a distinct experimental setup;
✓ Extension of the multiple linear regression to determine a robust mathematical relationship between the variables under investigation.

The multiple linear regression led to the extraction of the following analytical equation:

(2) Φ=−0.5641+0.5078×f+0.4345×G−0.0590×E

This model indicates a predominant linear relationship between the average S21 phase shift (Φ) and the frequency (f), with a positive and statistically significant influence of the maximum antenna gain (G) and a moderate effect of the average electric field strength (E). The linear model achieved a coefficient of determination R^2^ = 0.981, indicating a high predictive capability of the variation of the average phase shift over the entire 3 mm sweep of the target.

To verify the robustness and validity of our model, we used it across different simulated scenarios; a kind of “cross-validation” was applied. Simulations were made for f = 3 GHz, 12 GHz, and 17 GHz. The following phase shifts were obtained: 1.62 deg. 6.63 deg., and 14.32 deg.; the corresponding maximum gains (in linear scale) were 5.54, 9.3, and 16.8; the corresponding average values of the E-field strengths were 24.8 V/m, 14.16 V/m, and 16.58 V/m. According to the formula (2), we should have obtained 1.90 deg., 8.73 deg., and 14.38 deg., respectively. All of the checked scenarios provided proof of the robustness of the model.

**Figure 10:**
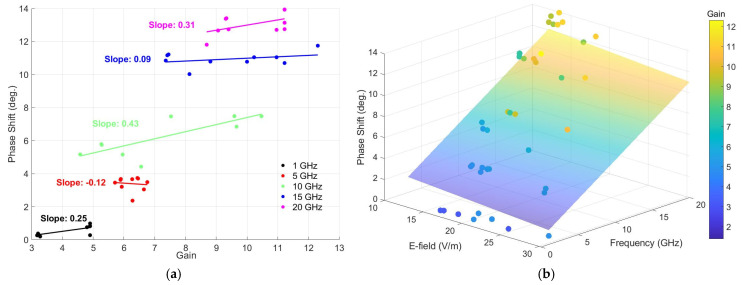
(**a**) The dependence of the average S21 phase shift on the antenna gain for each frequency (the slopes of the linear regression curves are shown); (**b**) a 3D model of the relationship between the average phase shift (recorded at a 3 mm displacement of the planar target), the frequency, the maximum antenna gain, and the average E-field strength on the target’s surface.

For a detailed analysis of the relationship between the antenna G and the mean phase shift Φ of the radar signal for each frequency, five additional linear regression analyses were performed. Each regression was performed separately for the 1 GHz, 5 GHz, 10 GHz, 15 GHz, and 20 GHz frequency, correspondingly. The results of these analyses are plotted in Figure 10a, where the slopes obtained for each frequency under investigation are explicitly indicated.

Analyzing the results presented in Figure 10a, it can be seen that the slope of the regression curve varies significantly with the frequency change. At frequencies of 1 GHz and 20 GHz, positive slopes were obtained (0.25 and 0.31), indicating a clear increase in the detection sensitivity of the radar system with an increasing antenna gain. For the 10 GHz frequency, the slope is positive and more pronounced (0.43), reflecting an increased sensitivity to gain variations. In contrast, for the 5 GHz frequency, the slope of the regression was negative (−0.12), suggesting that at this frequency, increasing the antenna gain does not lead to an improvement in the detection sensitivity. For the 15 GHz frequency, the moderate positive slope (0.09) indicates that the effect of the gain on sensitivity is remarkable, but less pronounced, compared to the 1 GHz and 20 GHz frequencies.

Figure 10b presents the 3D relationship between the phase shift, the average E-field strength, and the frequency. There is a pronounced increase in the S21 average phase shift with an increasing frequency, which confirms that electromagnetic waves experience a larger phase shift at the higher frequencies. The average E-field strength has a moderate effect on the phase shift, but the relationship is not linear. It is found that for higher values of E-field strength, the average phase increases more slowly, suggesting a saturation effect of the phase shift. At lower frequencies (below 5 GHz), the average phase shift over the 3 mm target path remains low, indicating that the reflections are more manageable and less affected by the electric field variations. Regarding the correlation between the phase, electric field strength, and gain, the higher average phase shift along the target travel generally corresponds to a stronger interaction between the field and the obstacle; copper, having an increased reflectivity, causes an additional phase shift relative to the muscle tissue. A higher electric field strength is associated with more effective reflection (in the case of copper), while the muscle tissue attenuates the incident wave more strongly.

The increased gain amplifies the differences in both the phase and field strength, making high directivity antennas more sensitive to any change in position. We observe a clear trend in which a higher antenna gain results in a higher average phase shift, suggesting an increased reception sensitivity to small variations in the position of the radar planar target. The more intensely colored dots (higher gain values) in Figure 10b are associated with larger average phase shifts, indicating a slight influence of the incident electric field strength on the phase shift. At high frequencies, the average phase shift increases even for moderate gains, suggesting that under these conditions, the phase shift is more frequency-dependent than gain-dependent.

**Figure 11:**
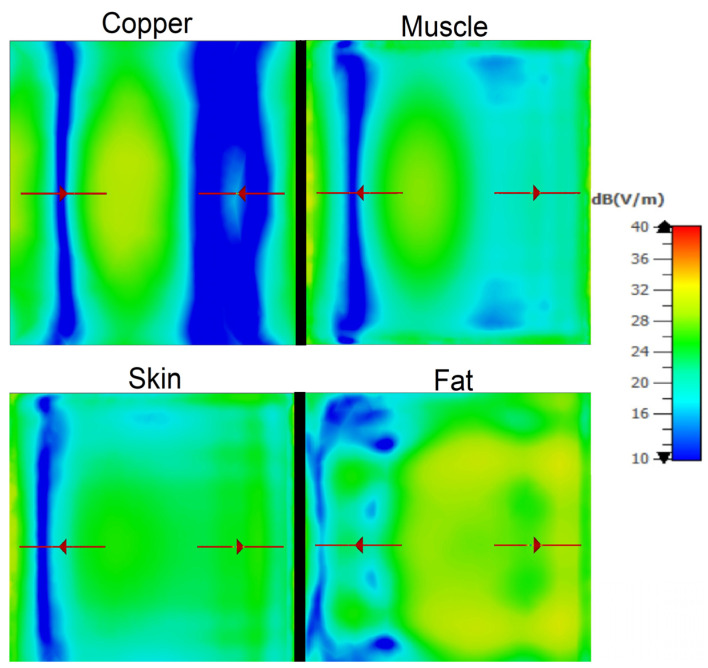
The distribution of the E-field strength on the surface of different materials composing the Doppler target (copper, muscle, skin, and fat adipose tissue) at 5 GHz in the same Doppler configuration.

Figure 11 illustrates the E-field strength distribution on the surface of several investigated materials: copper, muscle tissue, skin tissue, and fat tissue at 5 GHz. For the copper target, the E-field strength is more pronounced, indicating a high reflection efficiency, thus justifying the higher phase shifts previously observed for this material. In contrast, the muscle tissue exhibits a lower reflected E-field strength, which is due to the higher dielectric losses and higher attenuation specific to this biological material. The fat tissue and skin tissue show intermediate behaviors; the skin tissue more closely mimics the muscle tissue.

**Figure 12:**
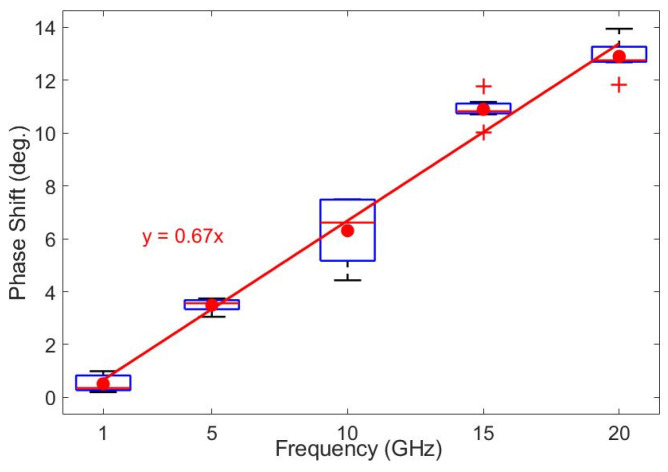
The boxplots of every 8 simulated cases per investigated frequency, showing the distribution of the S21 phase shift values while varying the considered independent variables; the linear regression emphasizes the greatest role of the used frequency in establishing the sensitivity of the CW radar to the minute movements of the target position.

Summarizing all of the 40 cases studied (see Figure 10a), we present the boxplot statistics marking the most important factor that influences the transmission phase shift change, namely the used frequency. As observed in Figure 12, almost all boxplots present small interquartile ranges (with the exception of 10 GHz), proving that the other tested independent variables have a lower influence. For the studied situation, a linear regression was conducted, and the slope of the curve of the phase shift dependence upon the frequency was 0.67, proving the very good prediction capability of our model based essentially on the frequency dependence of the Doppler radar sensitivity.

**Figure 13:**
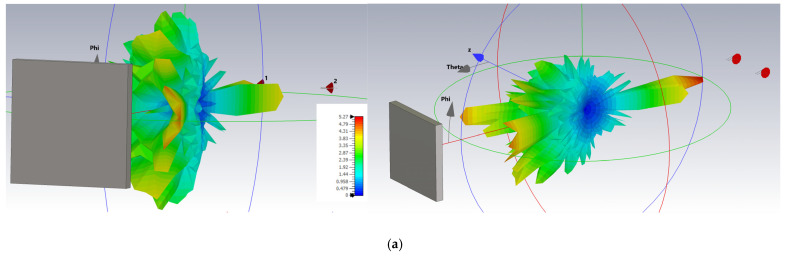

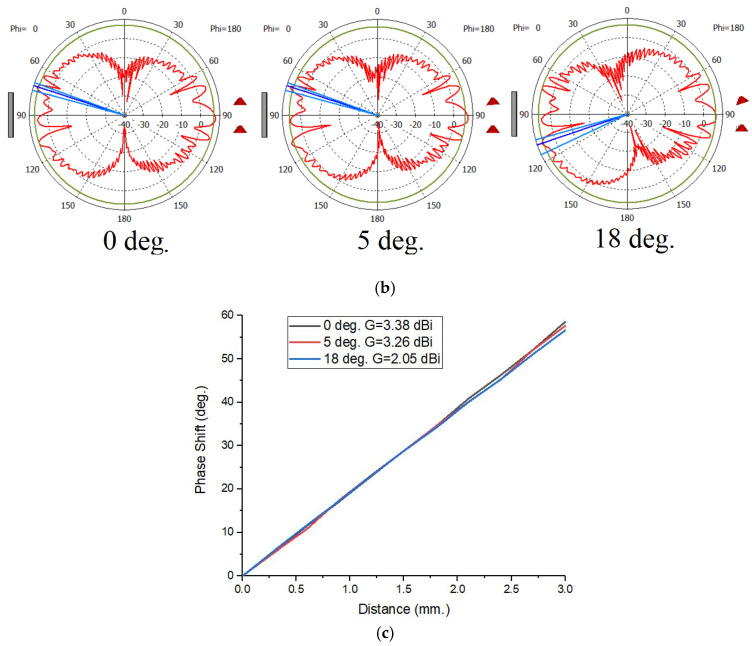
The effect of the rotation of the Yagi antenna plane against the target plane at 10 GHz (off-boresight effects): (**a**) the figuration of the 3D radiation pattern affected by the target existence; (**b**) the planar radiation pattern of the antenna at three different rotation angles; (**c**) the impact of the rotations and the perpendicular gain value on the S21 phase shift.

A supplementary analysis was performed at the end of the study, with the objective of studying the changes resulting from the off-boresight measurements for the high gain (Yagi) antenna. As shown in Figure 13a, the 3D radiation pattern (the gain is in linear scale) is generally dependent on the target rotative position in relation to the antenna plane, and the radiation lobes may suffer changes in the amplitude and position in space when the configuration suffers rotations. The distance between the target and the antenna was maintained at 500 mm. We assessed the effect of rotating the antennas from 0 to 5 and 18 degrees around the target. The rotation effect on the polar radiation pattern change in these three situations is indicated in Figure 13b. Finally, the effect on the S21 phase shift for the three rotations is shown in Figure 13c. The far-field condition and the (2.05–3.38) dBi gain range inspected ensure a very low impact from the off-boresight simulations on the phase shift results of the Doppler radar configuration.

Another aspect that could be addressed in a future study is the situation that might appear at lower frequencies (1–5) GHz, when the Doppler signals penetrate the tissue but are affected by moving structures beneath the surface of the body. The pulsatile blood flow and chest wall movements may induce small but measurable S21 phase shifts. These movements cause minute changes in the dielectric properties and tissue interfaces, leading to variations in the received waveform that may manifest as low-amplitude, non-sinusoidal components and harmonics. The assessment of such effects could be approached by simulations in CST Studio, but they were beyond the scope of the present research. In future work, we propose addressing this aspect by a step methodology, which should comprise the following: (1) developing a multi-layer tissue model representing skin, fat, muscle, and blood vessels and incorporating elements that simulate periodic subsurface motion—oscillating or vibrating structures within the tissue layers mimicking arterial pulses or chest movements; (2) running simulations for the static tissue model to establish a baseline S21 parameter and then introducing time-varying boundary conditions or dielectric property fluctuations within the tissue model to simulate movement; (3) performing frequency domain simulations to observe the phase shifts in S21 and time-domain simulations to capture the transient effects caused by subsurface motions; (4) analyzing the evolution of the phase of S21 over time to identify shifts correlated with simulated movements and using signal processing of the time-domain data to detect the low-level harmonics and non-sinusoidal components induced by the movement; (5) measuring the magnitude of the phase shifts and amplitude variations due to simulated movement and study how these variations fluctuate with parameters like frequency, distance, or movement amplitude; and (6) parameter sensitivity checking by varying the movement amplitude and frequency to assess how these factors influence the measured Doppler shifts. However, the discussed effects may affect only the low-frequency range, which was demonstrated not to be the best solution in the present work.

## 4. Conclusions

This study investigated the influence of the antenna parameters and frequency on the sensitivity of a CW Doppler radar system for detecting subtle motions associated with human breathing. Through computational simulations that covered 40 cases, we analyzed the S21 transmission coefficient’s phase shift variation in response to very small changes in the target position. The system configuration involved two identical antennas (for emission and reception) and a reflective planar target (mimicking a portion of the human chest) with variations in the target material (PEC, copper, muscle, skin, and fat) and antenna types (dipole and Yagi—two models with different gains).

Our findings reveal that the antenna type, operating frequency, gain, and directivity significantly influence (and in different degrees) the S21 phase shift and, consequently, the detection sensitivity. The Yagi antennas exhibited a more depressed effect at lower frequencies, while the dipoles showed effects that are more pronounced only at higher frequencies. The S21 phase shift was generally lower for the muscle tissue compared to the copper, indicating that lossy dielectric materials reduce the phase shift amplitude, proving that the target material also influences the detection sensitivity.

A multiple linear regression analysis revealed a predominant linear relationship between the average S21 phase shift and the frequency, with a positive influence from the antenna gain and a moderate effect from the electric field strength on the target’s surface. The linear model achieved a high predictive capability (R^2^ = 0.981) for the variation of the average phase shift. Positive slopes were obtained at frequencies of 1 GHz and 20 GHz, indicating a clear increase in the detection sensitivity with an increasing antenna gain.

The study also demonstrated that higher gain antennas are more sensitive to changes in the target material. The 5-element Yagi antenna provided a better separation of results between the different tissue types, making it more suitable for applications requiring accurate phase shift tracking. The use of a higher gain antenna and a higher frequency (above 10 GHz) provided the best sensitivity corresponding to differences between tissue types during the respiration monitoring.

In summary, this research provides valuable indications regarding the selection and use of antennas in CW Doppler bio-radar systems. The results highlight the importance of considering the antenna parameters, operating frequency, and target material properties to optimize the detection performance and sensitivity. These findings can be used to develop more effective Doppler bio-radar systems for various applications, including breath monitoring and fine motion detection.

## Figures and Tables

**Table 1:** The dimensions of the antennas and the minimum far-field distance from the antennas.

f [GHz]	Dmax_Dipole [mm]	L_Far field Dipole [mm]	Dmax_Yagi [mm]	L_far field Yagi [mm]
1	135.40	139.01	154.00	122.22
5	27.30	38.49	42.30	24.84
10	13.23	16.37	18.56	11.67
15	8.77	11.36	12.96	7.69
20	6.40	7.90	9.25	5.46

## Data Availability

The original contributions presented in this study are included in the article. Further inquiries can be directed to the corresponding author.
